# Immunization with nanovaccines containing mutated K-Ras peptides and imiquimod aggravates heterotopic pancreatic cancer induced in mice

**DOI:** 10.3389/fimmu.2023.1153724

**Published:** 2023-04-12

**Authors:** Amparo Martínez-Pérez, Lara Diego-González, Manuel Vilanova, Alexandra Correia, Rosana Simón-Vázquez, África González-Fernández

**Affiliations:** ^1^ CINBIO, Universidade de Vigo, Inmunology Group, Vigo, Spain; ^2^ Instituto de Investigación Sanitaria Galicia Sur (IIS Galicia Sur), SERGAS-UVIGO, Vigo, Spain; ^3^ I3S-Instituto de Investigação e Inovação em Saúde, Universidade do Porto, Porto, Portugal; ^4^ IBMC-Instituto de Biologia Molecular e Celular, Universidade do Porto, Porto, Portugal; ^5^ ICBAS-Instituto de Ciências Biomédicas de Abel Salazar, Universidade do Porto, Porto, Portugal

**Keywords:** KRAS, nanovaccine, pancreatic cancer, imiquimod (IMQ), chitosan, polyarginine

## Abstract

**Purpose:**

The growing incidence and lethality of pancreatic cancer urges the development of new therapeutic approaches. Anti-tumoral vaccines can potentiate the immune response against the tumor, targeting specific antigens expressed only on tumor cells. In this work, we designed new vaccines for pancreatic cancer, composed by chitosan nanocapsules (CS NCs) containing imiquimod (IMQ) as adjuvant, and targeting the K-Ras mutation G12V.

**Experimental design:**

We tested the immunogenicity of our vaccines in mice, carrying different combinations of K-Ras mutated peptides. Then, we analyzed their prophylactic and therapeutic efficacy in mice bearing heterotopic pancreatic cancer.

**Results:**

Unexpectedly, although good results were observed at short time points, the different combinations of our CS NCs vaccines seemed to potentiate tumor growth and reduce survival rate. We propose that this effect could be due to an inadequate immune response, partially because of the induction of a regulatory tolerogenic response.

**Conclusion:**

Our results call for caution in the use of some NCs containing IMQ in the immunotherapy against pancreatic cancer.

## 1 Introduction

Pancreatic ductal adenocarcinoma (PDAC) is one of the deadliest types of cancer, with poor prognosis due to the difficulty of an early diagnosis (1, 2). Several types of cancer, including PDAC, often accumulate mutations in the KRAS oncogene. The mutations commonly lead to a permanently activation of the protein, which ultimately promotes tumor transformation and proliferation. KRAS mutations are found in around 90% of PDAC cancers, often involving codon 12 (3, 4).

For a long time, mutations in the Ras family have been prioritized as therapeutic targets (5). These approaches include the development of anti-tumoral vaccines that could help in the recognition and further attack against the malignant cells by the immune system. Although vaccines targeting K-Ras mutations had reached clinical trials, they have shown modest benefits (reviewed in (6)). Some strategies have combined K-Ras vaccination with classic chemotherapy or used it after after tumor resection surgery.

The development of new vaccines and adjuvants offers novel possibilities for either therapeutic or preventive cancer vaccines. Nanoparticles have been successfully proven as good vaccine delivery systems due to their properties: 1) they can encapsulate antigens and adjuvants, protecting them from premature degradation; 2) their characteristics at the nanoscale (size, shape, charge) and similarity to pathogens. They can facilitate the uptake by antigen-presenting cells (APCs), allowing good immunogenic properties (7); and 3) they can easily drain to the closest lymph nodes from the administration site, including the tumor-draining lymph nodes (8).

Moreover, the incorporation of additional immunostimulants could improve the vaccine immunogenicity. Imiquimod (IMQ) is used in creams as a drug to treat precancerous growths (like actinic keratosis) and genital warts on adults. IMQ interacts with Toll-like receptor 7 on innate immune cells, inducing the expression of cytokines favoring a Th1-type immune response. IMQ can act as adjuvant in vaccines for infectious diseases (9, 10), but also in anti-tumoral vaccines, increasing the immunogenicity of a tumor-lysate vaccine against T cell lymphoma (11), or melanoma (12), among others. However, some publications reported opposite effects (13), presumably due to the specific induction of immunosuppressive reactions.

In our study, we designed a chitosan-based nanovaccine carrying K-Ras G12V mutated peptides, and IMQ as adjuvant, included in the oily core of the nanocapsule. We assessed its immunogenicity and tested its efficacy either as a preventive or therapeutic vaccine against PDAC in mice. Furthermore, we compared our vaccine with another K-Ras-mutated vaccine in clinical trials, TG02 (from the Targovax company), that incorporates granulocyte macrophage colony-stimulating factor (GM-CSF) as adjuvant, either used alone or in combination with our nanovaccine.

## 2 Materials and methods

### 2.1 K-Ras mutated peptide design

Peptides from the K-Ras mutated protein were designed using Immune Epitope Database (IEDB) major histocompatibility complex (MHC) binding prediction tool (http://tools.iedb.org/mhci/), taking into account the class I and class II MHC molecules from the mouse strain to be used (C57BL/6) and the K-Ras mutated sequence (G12V). Peptides with the lowest percentile rank to C57BL/6 were selected for MHC-II (YKLVVVGAVGVGKSA peptide p1, KLVVVGAVGVGKSAL peptide p2) and MHC-I (AVGVGKSAL peptide p3). Mutated K-Ras peptides will be hereafter referred to as Krasm. More information about peptides can be found in **Table S1**.

### 2.2 Nanovaccines’ synthesis

Polymeric chitosan nanocapsules (CS NCs) or inulin/polyarginine nanocapsules (INU/pArg NCs) with IMQ (14) as adjuvant were synthetized as described previously (10). Mixture of the NCs with the peptides were prepared just before each usage in a 1:1.5 ratio (polymer:antigen). The mixture was incubated with constant orbital shaking (300 rpm) at room temperature (RT) for one hour to allow antigen adsorption to the polymer surface. In this way, 10 µg of IMQ and 200 µg of peptides were added per dose. Estimated IMQ encapsulation efficiency was 60–70% (10). Peptide absorption on the nanocapsule surface was 50-60%, determined by liquid chromatography–mass spectrometry (LC-MS), and similar to the antigen adsorption previously described for an intranasal vaccine (10). Following the same procedure, antigen not bound to the NCs was not removed from the suspension. Complete CS and INU/pArg nanovaccines containing the peptides will be referred to as CS-Krasm and INU/pArg-Krasm, respectively.

In addition to our CS-Krasm, we also worked with a commercial vaccine, TG02, belonging to the company TARGOVAX ASA (Olso, Norway). It is a cocktail of 8 peptides of 17 amino acids with the K-Ras G12V mutation of interest, apart from other classic mutations of this protein at that point. The peptide sequences are KLVVVGAG**C**VGKSALTI and KLVVVGA**V**GVGKSALTI (amino acid indicated in bold can be a C or a D in the first one and A, C, D, R, S or V in the second one (https://patents.google.com/patent/EP3140320B1/en).

### 2.3 Cell culture

Pancreatic tumor cells ATQ303G were gently provided by Dr. Carmen Guerra and Prof. Mariano Barbacid from Centro Nacional de Investigaciones Oncológicas (CNIO, Madrid, Spain). Cells were generated from PDAC tumors developed by Elastase-tTA; Tet-O-Cre; K-Ras+/LSLG12Vgeo; p53lox/lox mice (C57BL6/129 background) described in (15). Pancreatic cells were cultured in presence of DMEM High Glucose + Glutamax (Gibco; Waltham, MA, USA).

THP-1 and Jurkat cell lines were purchased from the American Type Culture Collection (ATCC) (VA, USA), while human peripheral blood mononuclear cells (hPBMCs) were obtained from three healthy donors by isopycnic or density gradient centrifugation (Ficoll, GE Healthcare; Boston, MA, USA) from whole blood. For these cells, RPMI (Roswell Park Memorial Institute) 1640 culture medium (Corning; Corning, NY, USA) was used.

In all cases, culture medium was supplemented with 10% Fetal Bovine Serum (Sigma-Aldrich; Burlington, MA, USA) and 1% Penicillin-Streptomycin (Gibco). Cells were maintained at 37°C and 5% CO_2_.

### 2.4 Apoptotic assay

Cytotoxicity of NCs was determined by flow cytometry using the apoptosis detection kit (Immunostep S.L; Salamanca, Spain) by double labeling Annexin V-FITC and Propidium Iodide (PI). The following cell populations were determined: Annexin V^–^ IP^–^ (live cells), Annexin V^+^ IP^–^ (early apoptotic cells), Annexin V^+^ IP^+^ (late apoptotic cells) and Annexin V^–^ IP^+^ (necrotic cells).

For this, 100,000 Jurkat or hPBMCs cells/well were seeded in flat-bottom 96-well plates (Costar, Corning). 24 hours later, NCs were added at 50 or 100 µg/mL. After 24 hours of incubation, cells were collected and labeled with Annexin V-FITC and IP according to the protocol described by the manufacturer. Cells were read in the FC500 cytometer (Beckman Coulter, IN, USA) and data analysis was performed using FlowLogic™ software (version 7.1, Miltenyi Biotec; Bergisch Gladbach, Germany).

### 2.5 xCELLigence assay

ATQ303G cells were seeded at a density of 7,500 cells/well in special 16-well plates with gold electrodes from the xCELLigence^®^ RTCA DP Instrument system (Roche Diagnostics, version 1.2.1). The equipment measures impedance to monitor cell growth and cell death in real time. It transforms the electrical signal into numerical values represented as a Cell Index. Cells were kept in culture until reaching the exponential phase of growth. Once achieved, 100 µg/mL of CS NCs were added. Untreated cells and culture medium alone are used as controls. The Cell Index was normalized to the time of NC addition, and the percentage of cell capacity was normalized with respect to the negative control.

### 2.6 Hemolysis

Blood from healthy mice or humans was obtained by cardiac or intravenous puncture, respectively, and treated with heparin. Blood was diluted to 3% in PBS (w/v) and 80 µL of this erythrocyte suspension was incubated with 80 µL of the NCs (50 and 100 µg/mL). PBS was used as negative control and 1% Triton X-100 in PBS was used as positive control. After 4 hours of incubation at 37°C, cells were centrifugated at 1000 x gg for 10 min at 4°C. 80 µL of the supernatant were transferred to a new plate to read the optical density (OD) at 558 in the multidetector EnVision (Perkin-Elmer Inc.; Norwalk, CT, EEUU).

The hemolytic percentage was calculated assuming that PBS is 0% and PBS-Triton X-100 is 100% from the following formula “hemolysis (%) = [(OD sample – OD PBS)/(OD Triton – OD PBS)] × 100”. Per the protocol of the American Society for Testing and Materials (ASTM) International E2524-08 hemolysis percentages above 5% were considered positive. Percentages between 5 and 2% are considered as partially hemolytic, while percentages lower than 2% were considered non-hemolytic (16).

### 2.7 Coagulation assay

It was carried out according to the Method ITA-12 V1.1 protocol (Laboratory NC. Assay Cascade Protocols. Available: http://ncl.cancer.gov/working_assay-cascade.asp) by determining the prothrombin time (PT), the activated partial thromboplastin time (APTT) and thrombin time (TT).

Blood from several healthy donors was obtained by intravenous puncture and in a tube with sodium citrate as an anticoagulant. The plasma was recovered after centrifugation of the blood for 10 min at 2500 x gg and 21°C. 900 µL of pooled plasma were incubated for 30 min at 37°C with 100 µL of the NCs diluted in PBS at three concentrations (25, 50 and 100 µg/mL). PBS was used as a negative control. After the incubation time, the reading was carried out in the Start 4 coagulometer (Diagnostica Stago; Parsippany, NJ, USA) by adding the corresponding coagulation-inducing reagents. **Table S2** shows the conditions used.

### 2.8 Macrophage polarization

The THP-1 monocytic cell line was used for this experiment. 400,000 cells/well were plated in a 48-well plate (Costar) with a final phorbol 12-myristate 13-acetate (PMA, Abcam) concentration of 10 ng/mL for their differentiation into macrophages. After 72 hours, the treatments for specific phenotype differentiation (20 ng/mL of IFN-γ (Miltenyi Biotec) + 5 ng/mL of lipopolysaccharide (LPS) (InvivoGen; San Diego, CA, USA) for the M1 phenotype and 20 ng/mL of IL-4 (Sigma-Aldrich) + 10 ng/mL de M-CSF (Miltenyi Biotec) for the M2 phenotype) were added for 24 hours. Then, 100 µg/mL of CS NCs were added. The culture medium was used as negative control. After 24 hours of the addition of the different treatments, cells were harvested and centrifugated at 1200 rpm for 5 min. Then, cells were resuspended in 10 µL of FcR blocking reagent (Immunostep S.L) and incubated for 15 min at RT. Next, 5 µL of the following antibodies were added: anti- CD206, CD163, HLA-DR, CD86 and CD14 (**Supplementary Table S3**). Cells were incubated with the antibodies for 30 min at 4°C. Subsequently, they were washed and resuspended in PBS for analysis by flow cytometry (Cytoflex S, Beckman Coulter). Data analysis was performed with the CytExpert analysis program (Beckman Coulter, version 2.3.1.22).

### 2.9 Mice

Seven-week-old C57BL/6JRj female mice were purchased from Janvier Labs (Le Genest-Saint-Isle, France) (or C57BL/6J imported from Charles River to i3s Animal Facility of Instituto de investigação e inovação em saúde, University of Porto, specifically for one experiment). Mice were maintained under specific pathogen-free conditions at the animal facility of Servizo de Bioexperimentación Universidade de Vigo, Spain (or i3s Animal Facility in one experiment), with constant temperature (20 ± 1°C) and humidity (55 ± 10%). The animals were fed with sterile water and diet water *ad libitum* under standardized light-controlled conditions (12 h light and dark periods). Mice were acclimatized for at least 1 week before use. Animal experiments were performed with ethical approval from the hosting institutions and according to the national regulations and legislation of that country.

### 2.10 Vaccination

Mice were vaccinated three times separated 21 days. 100 µL of prepared vaccines were subcutaneously administered per injection in the left flank. Mice vaccinated with our vaccines received a total of 200 µg of the peptide mixture (three peptides), subcutaneously in the left flank. Mice vaccinated with TG02 vaccine received 800 µg of the peptide mixture subcutaneously in the left flank and 2.5×10^5^ U of GM-CSF 15-30 min later.

### 2.11 Tumor induction and monitoring

Mice were anesthetized with isoflurane and 3×10^6^ cells in PBS: Matrigel (Corning) (1:1 proportion) were injected subcutaneously in the right flank (100 µL). Mice were supervised and weighed every day, and tumor diameter was measured with a caliper. Tumor volume was calculated using the formula “volume = (wide^2^ × length)/2”.

### 2.12 IgG antibody specific ELISA

Peptides were coupled to Bovine serum albumin (BSA) protein (VWR; Radnor, PA, USA) to improve their attached to plastic plates and favor the detection of peptide-specific antibodies in ELISA. 2 mM N-(3-Dimethylaminopropyl)-N′-ethylcarbodiimide hydrochloride (Sigma-Aldrich) and 5 mM N-Hydroxysulfosuccinimide sodium salt (Sigma-Aldrich) were added to BSA 1 mg/mL and reacted for 15 minutes at RT. Then, p1, p2 and p3 peptides were added in a 15:1 peptides:BSA ratio (5:1 for each peptide) and reacted for 2 hours at RT.

ELISA plates (Thermo Fisher Scientific; Waltham, MA, USA) were coated with BSA-peptides conjugated for 3 hours at RT. Then, plates were blocked with PBS 1% BSA at 4°C overnight. Diluted serum was incubated for 1 hour at RT. Wells were washed three times with PBS-Tween 0.05% and incubated with secondary antibodies: goat anti-mouse Igs linked to horseradish peroxidase (HRP) (1:5000) (BioRad; Hercules, CA, USA). Wells were washed three times and TMB substrate (3,3’,5,5’-Tetramethylbenzidine) was added for 15 min at RT. Reaction was stopped by adding H_2_SO_4_. Optical density (OD) was measured at 450 nm using an Envision Multilabel Plate Reader (Perkin Elmer; Waltham, MA, USA).

To eliminate background noise, the OD value from blank wells (incubated with PBS) was subtracted. OD values from wells coated with BSA but without peptides, were also subtracted from the corresponding OD of peptide-coated wells.

### 2.13 Tumor infiltrating immune fraction analysis

Mice were sacrificed with CO_2_ and subcutaneous tumors were extracted. Half tumor was processed to study immune infiltrating fraction. Tumors were cut with a scalpel into pieces of 2-3 mm in diameter and incubated with 1 mg/mL Collagenase (Gibco) at 37°C for 30 min. Then, tumors were homogenized and filtered through a 100 mm nylon mesh cell. Immune fraction was isolated using density gradient Ficoll Premium 1084 (GE Healthcare; Chicago, IL, USA) and centrifuged at 1800 rpm for 30 min at 22°C. Immune cells were washed and stained for flow cytometry.

Tumor infiltrating cells were incubated with Fixable Viability Stain 510 and antibodies anti-CD45, CD3, CD4, CD8, NK1.1 and MHC-II for 30 min at 4°C. Then, cells were fixed and permeabilized using mouse Foxp3 fixation buffer (BD Pharmigen; San Diego, CA, USA) for 30 min 4°C in the dark and permeabilized using mouse Foxp3 permeabilization buffer (BD Pharmigen) 30 min 37°C in the dark. Next, cells were stained with anti-Foxp3 antibody and fixed with paraformaldehyde (PFA) 2% for 30 min at 4°C. Samples were run on an CytoFLEX S flow cytometer (Beckman Coulter; Brea, CA, USA), and data were analyzed using CytExpert (version 2.3.1.22, Beckman Coulter) (or FlowLogic (version 7.1, FlowLogic; UK) for one experiment) software. A list of antibodies used, and references can be found in **Table S2**.

### 2.14 Splenocytes analysis

Spleens were homogenized and filtered through a 100 mm nylon mesh cell. Red blood cells (RBC) were lysed with 3 mL of RBC lysis buffer for mouse (Alfa Aesar, Thermo Fisher Scientific) for 4 min. Then, cells were washed with RPMI 1640 (Corning) media supplemented with 10% Fetal Bovine Serum (Sigma-Aldrich) 1% Penicillin-Streptomycin (Gibco) 50 nM β-mercaptoethanol (Sigma-Aldrich) and 10 mM Hepes (VWR).

Splenocytes from tumor-bearing mice were processed and kept in liquid nitrogen to be plated and analyzed simultaneously. Cells were stored in a cryopreserving solution (Fetal Bovine Serum with 10% Dimethyl sulfoxide (Sigma-Aldrich) and kept at –196°C in liquid nitrogen until further use. Live cells were counted and 5×10^5^ splenocytes per well were plated in a 96-well plate. Cells were stimulated with a final concentration of 100 µg/mL of either 3-peptide-mixture or 100 µg/mL of TG02-peptide-mixture at 37°C with 5% CO_2_ for 2 days.

During the last 4 hours of stimulus, 10 µg/mL of Brefeldin A (Sigma-Aldrich) were added to block Golgi apparatus protein processing. Cells were then centrifuged, and supernatants were kept for cytokine analysis. Splenocytes were incubated with Fixable Viability Stain 510 and antibodies anti-CD3, CD4, CD8, CD25, CD62L and CD44 for 30 min at 4°C. Then, cells were fixed using mouse Foxp3 fixation buffer (BD Pharmigen) for 30 min 4°C in the dark and permeabilized using mouse Foxp3 permeabilization buffer (BD Pharmigen) 30 min 37°C in the dark. Next, cells were stained with anti-Foxp3, -IFN-γ and -IL-4 antibodies in Brilliant Stain Buffer (BD Pharmigen) for 30 min at 4°C. Cells were then washed and fixed with paraformaldehyde (PFA) 2% for 30 min at 4°C. Samples were run on an CytoFLEX S flow cytometer (Beckman Coulter) (or BD FACSCanto™ II for one experiment), and data were analyzed using CytExpert (version 2.3.1.22, Beckman Coulter) (or Flowlogic (version 7.1, FlowLogic; UK) for one experiment) software. A list of antibodies used and references can be found in **Table S3**.

### 2.15 Multiplex cytokine assay

Supernatants from splenocyte cultures were used for cytokine detection using the customized MILLIPLEX MAP kit Mouse Th17 Magnetic Bead Panel (GM-CSF, IFN-γ, IL-1β, IL-2, IL-4, IL-6, IL-10, IL-12p70, IL-17A, TNF-α) (Merk; Kenilworth, NJ, USA). Immunoassay was performed following manufacturer’s instructions. Samples were analyzed in triplicates with a MAGPIX reader (Luminex; Austin, TX, USA) and the results were analyzed using the xPonent 4.2 software.

### 2.16 TNF-α assay for assessing trained immunity.

Peritoneal macrophages were extracted from C57BL/6 mice. Mice were sacrificed with CO_2_ and 5 mL of RPMI 1640 media (Corning), supplemented with 10% Fetal Bovine Serum (Sigma-Aldrich) and 1% Penicillin-Streptomycin (Gibco), was injected into the peritoneal cavity. The abdomen was gently massaged for 5 minutes to dislodge attached macrophages. Then, peritoneal fluid was recovered using a Pasteur pipette.

5×10^5^ macrophages per well were seeded on 96-well plates and let rest for 2 hours at 37°C 5% CO_2_. CS NCs were added at 20 µg/mL (or free media) and incubated overnight. Conditions were tested in biological duplicates. Media was replaced every two days. At day 7, macrophages were incubated with 0.1 µg/mL LPS (Sigma-Aldrich) (or free media) for 3 hours. Then, cells were incubated with new media overnight. At day 8, cells were centrifuged and supernatants recovered.

Tumor necrosis factor α (TNF-α) levels were measured using Mouse TNF alpha Uncoated ELISA kit (Invitrogen), following manufacturer’s instructions. TNF-α standard control was used to determine cytokine levels. OD was measured at 450 nm using an Envision Multilabel Plate Reader (Perkin Elmer).

### 2.17 Statistical analysis

Statistical analyses were performed using the GraphPad Prism 8 software. A Shapiro-Wilk test was carried out to determine the normal distribution of the samples. Statistical tests used are specified in each experiment description.

## 3 Results

### 3.1 Characterization of CS-nanocapsules

#### 3.1.1 Physicochemical characterization

CS NCs were designed following the procedure previously described (10), containing IMQ at the oily core. The antigen selected corresponds to a combination of 2 or 3 peptides containing the K-Ras G12V mutation (**Supplementary Table S1**, **Figure S1A**). CS NCs, both alone and with incorporated peptides, were shown to be homogeneous with a polydispersity index (PDI) ≤ 0.1, a mean size of 178 nm and positive charge (**Table 1**).

**Table 1:** Physical-chemical characterization of nanocapsules of CS in the absence and presence of mutated antigenic peptides.

NCs	Size (nm)	ζ (mV)
CS	179.8 ± 8.10	(+) 27.6 ± 5.20
CS+3p	167.7 ± 0.99	(+) 18.4 ± 0.99
CS+TG02	186.5 ± 5.00	(+) 19.6 ± 0.5

CS: chitosan; NCs: nanocapsules; TG02: Targovax vaccine; ζ: zeta potential; 3p: combination of the three mutated peptides K-Ras G12V.

#### 3.1.2 Biological characterization

Previous data indicated that CS NCs were cytocompatible on macrophages and lung epithelial cells and induced the production of reactive oxygen species (ROS) at long incubation times, the release of cytokines (mainly TNF-α and IL-6), and were able to activate the complement system, which could favour the vaccine efficacy (10). Here, the assessment of the cytotoxicity was expanded by studying their potential harmful effect on different immune cells. The Jurkat cell line (immortalized human T lymphocyte cells) and human peripheral blood mononuclear cell (hPBMCs) were incubated with CS NCs at two different concentrations (100 and 50 µg/mL) for 24 hours. As expected, CS NCs showed to be cytocompatible in both cell types at the concentrations tested (**Supplementary Figure S1B**).

The interaction with blood cells-components was assessed by hemolytic assay and coagulation time. For hemolysis assay, unwashed whole blood was used to mimic the most physiological environment possible (17). In mouse erythrocytes CS NCs did not induce hemolysis (< 2%), except at the highest concentration tested (100 µg/mL), with a hemolytic percentage of 4.75 ± 0.8, indicative of partial hemolytic activity (<5%). These results were similar to the data obtained with other CS NPs (18). The toxic effect of CS NCs was also tested on human blood from three healthy donors. In this case, CS NCs did not reach hemolytic levels at none of the concentrations tested (100 and 50 µg/mL) (**Supplementary Figure S1C**). As it can be observed in **Supplementary Figure S1D**, the CS NCs did not induce effects on any of the coagulation times analysed: prothrombin time (PT), activated partial thromboplastin time (APTT) and the thrombin time (TT). This indicates that CS NCs do not interfere with the coagulation cascade.

### 3.2 Immunization with CS-Krasm induces immunogenic response

To test the immunogenic effect of the vaccines, C57BL/6 mice were vaccinated three times at 21-day intervals. Mouse groups received PBS (control), CS (empty CS NCs), CS+2p (CS NCs containing Krasm peptides p1 and p2) or CS+3p (CS NCs containing the combination of the three Krasm peptides p1, p2 and p3). The vaccines were proven to be safe and not to induce weight loss on mice along the experiment.

**Figure 1:**
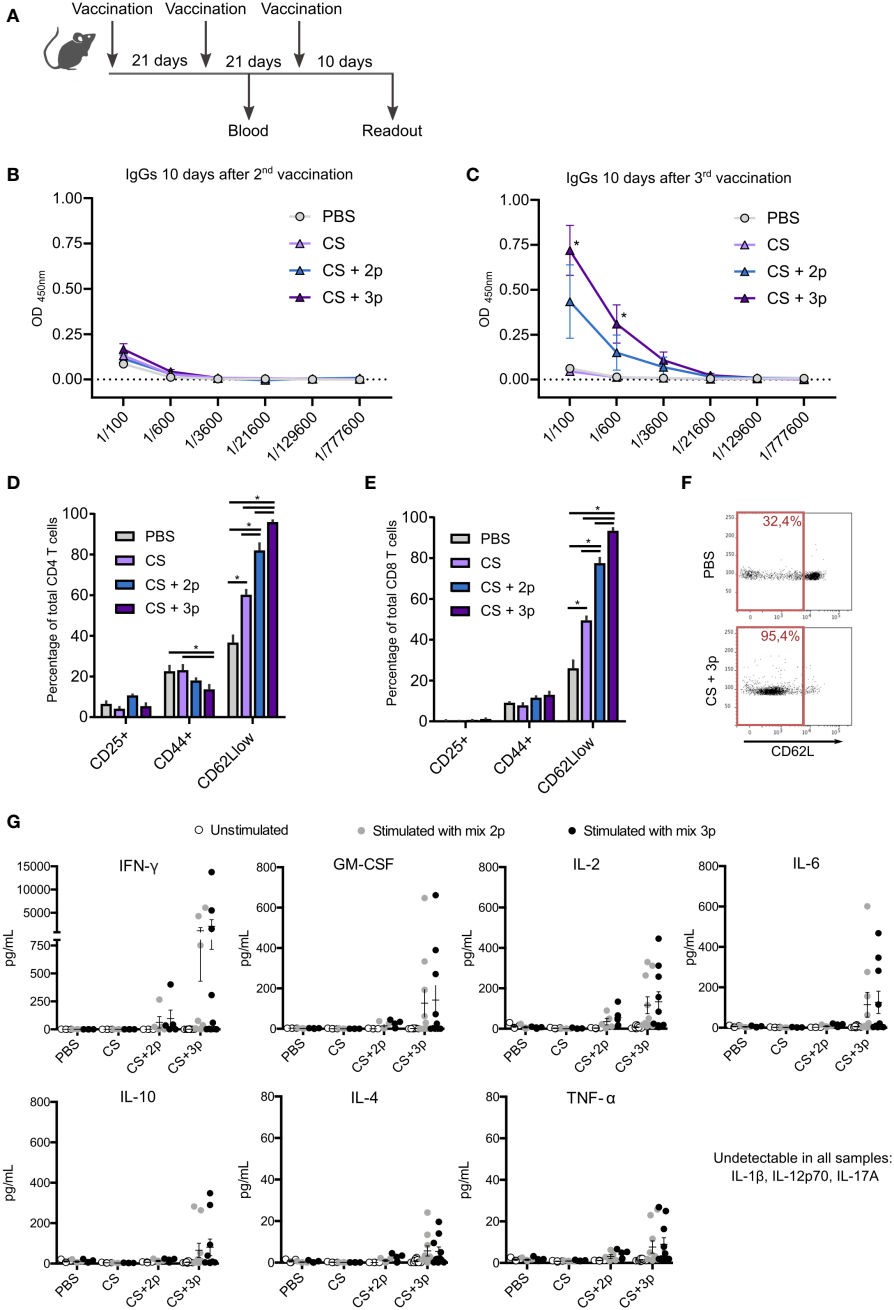
Characterization of the immune response generated by CS-Krasm vaccines. **(A)** Vaccination regimen is depicted. C57BL/6 mice 8-weeks old were vaccinated three times at 21-day intervals (n=5-10 per group). Blood was collected 10 days after 2^nd^ and 3^rd^ immunization. Ten days after 3^rd^ immunization, mice were sacrificed, and spleens were collected. The levels of specific IgGs that recognize a mixture of Krasm peptides (peptide 1, peptide 2 and peptide 3) were determined by ELISA immunoassay using serum collected ten days after the 2^nd^ **(B)** and 3^rd^ **(C)** immunization. Splenocytes were cultivated for 72 hours with the 3-peptides mixture. Then, lymphocyte biomarkers were evaluated by flow cytometry in CD4 T **(D)** and CD8 T cells **(E)**. Kruskal–Wallis and Dunn’s multiple comparison test were used for statistical analysis. *p < 0.05. **(F)** Representative example of the flow cytometry dot plot showing CD62L expression in PBS-vaccinated mice and CS+3p-vaccinated mice, with their respective percentages of CD62L low-expressing cells from ancestor gate. **(G)** Splenocytes were left unstimulated or incubated with either the 2-peptides mixture (peptide 1+ peptide 2) or the 3-peptides mixture (peptide 1 + peptide 2 + peptide 3) for 72 hours. Supernatants were collected and levels of cytokines GM-CSF, IFN-γ, IL-2, IL-4, IL-6, IL-10, TNF-α, IL-1β, IL-12p70 and IL-17A were measured using a multiplex cytokine assay.

Blood was collected 10 days after 2^nd^ and 3^rd^ immunization (**Figure 1A**) to analyze the existence of specific IgGs that recognize Krasm peptides. No significant differences among groups were found after the two first immunizations (**Figure 1B**). Nonetheless, specific antibodies were found after the third boost in both CS+2p and CS+3p-vaccinated mice, with significantly higher levels in the group receiving CS+3p (**Figure 1C**).

After their incubation with the 3-peptides mixture, T cells obtained from mice vaccinated with CS+2p or CS+3p showed a higher percentage of CD62L^low^ lymphocytes, indicative of effector T cells, in both CD4^+^ and CD8^+^ subtypes (**Figures 1D–F**). Intriguingly, CD4^+^ cells had a lower proportion of activated CD44^+^ cells elicited by groups CS+2p and CS+3p (**Figure 1D**). No differences among vaccination groups were found in the percentage of CD25^+^ T lymphocytes. Cells were also incubated with the individual peptides or left unstimulated, but no remarkable differences were found among culture conditions (**Supplementary Figure S2**).

Cytokine levels were measured in supernatants of cultured splenocytes stimulated with either the mixture of peptides p1+p2, or the mixture of the three peptides. Mice vaccinated with CS+3p produced the highest levels of cytokines, including GM-CSF, interferon-γ (IFN-γ), interleukin (IL)-2, IL-4, IL-6, IL-10 and tumor necrosis factor-α (TNF-α) (**Figure 1G**). Markedly lower levels of these cytokines were detected in the supernatants of splenocytes from mice vaccinated with the prototype CS+2p. The cytokines IL-1β, IL-12p70 or IL-17A were not detected in any case. Moreover, unstimulated cells generated no detectable levels of cytokines in any analyzed group.

Overall, our results demonstrate that both prototypes CS+2p and CS+3p induced in C57BL/6 mice the production of antibodies recognizing the Kras-mutated peptides and an immunogenic cellular response. However, prototype CS+3p seemed to induce the best immunogenic response.

### 3.3 Prophylactic effect of CS-Krasm vaccines

Based on the immunogenic evaluation, we selected the vaccine candidate CS+3p to test its possible preventive anti-tumoral effect and TG02 (from Targovax company) as control vaccine. As mentioned above, the TG02 vaccine contains a mixture of mutated peptides in combination with GM-CSF. We aimed to compare the anti-tumoral effect of our vaccine with that one induced by TG02, but also to test its efficacy combined with our CS nanoparticle formulation. Thus, several mouse groups followed different vaccination regimens, receiving either PBS, CS, CS+3p, TG02, CS+TG02 or CS+3p+TG02 (**Supplementary Figure S3A**). Eight days after vaccination, the pancreatic tumor cells were injected in one flank, and tumor growth was daily monitored. Mice were sacrificed following ethical endpoints.

The first 30 days after tumor injection, all vaccination groups showed the same survival rate (**Supplementary Figure S3B**). Between days 40 and 50, the percentage decreased in all groups, except for mice vaccinated with the combination CS+3p+TG02. This group remained with a 100% of viability for over 60 days.

We continued monitoring tumor growth until day 110 after tumor injection. At the end of the experiment, we observed that the highest survival ratios were obtained in the TG02-vaccinated group, whereas the remaining groups reached less survival than the control group. We analyzed for the presence of specific IgGs directed against Krasm peptides in blood at the moment of sacrifice. Nonetheless, no significant differences were found among vaccination groups (**Supplementary Figure S3C**).

The systemic immune response was also assessed through the culture of splenocytes, collected at the endpoint-sacrifice. Cells were cultured with antigens for 48 hours and further evaluated by flow cytometry. In this case, no phenotypic changes in the spleen immune populations were found among the different groups (**Supplementary Figure S3D**).

### 3.4 Therapeutic CS-Krasm vaccines do not prevent tumor growth, but rather favor it

We assessed whether the vaccines would have any beneficial effects when administered after tumor induction. This setting simulates better the most plausible scenario in the clinic: the administration of a therapeutic vaccine after tumor diagnosis.

**Figure 2:**
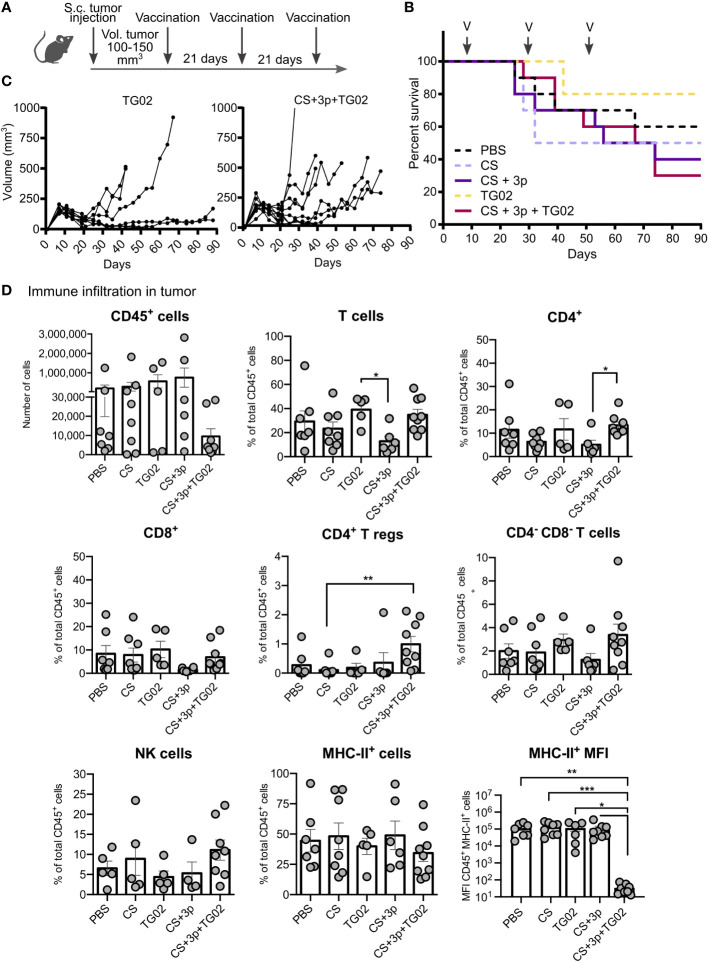
Effect of CS-Krasm used as therapeutic-vaccination in tumor-bearing mice. **(A)** Experimental protocol is depicted. C57BL/6 mice 8-weeks old were subcutaneously injected ATQ303G pancreatic cells in the left flank. When tumors reached a volume of 100-150 mm^3^, mice were vaccinated three times at 21-days intervals (n=10 per group). Blood was collected ten days after 2^nd^ and 3^rd^ immunization. Whenever mice reached their ethical time-point, mice were sacrificed, with blood and spleens collected. **(B)** Mice percentage of survival from day 0 (tumor injection) to 90. Vaccination days are indicated with an arrow and a V. **(C)** Tumor volume of TG02 and CS+3p+TG02 -vaccinated groups during the experiment. **(D)** Analysis of the immune infiltrate in tumors by flow cytometry. Data is represented in terms of total number of cells (CD45 biomarker), in percentage of total CD45^+^ leukocyte fraction or representing median fluorescence intensity (MFI) values for marker MHC-II in CD45^+^MHCII^+^ population (MHC-II MFI graphic). Kruskal–Wallis test and Dunn’s multiple comparison test were used for statistical analysis. *p < 0.05; **p < 0.01, ***p < 0.001.

Pancreatic tumor cells were subcutaneously injected and, when tumors reached a volume of 100-150 mm^3^, the first vaccine dose was administered. Mice were vaccinated following the same regimen with either PBS, CS, CS+3p, TG02 or CS+3p+TG02 (**Figure 2A**; **Supplementary Figure S4**).

The first 20 days after tumor injection, all vaccination groups shared the same survival rate (**Figure 2B**). Around day 40, TG02 and CS+3p+TG02 groups displayed again the best survival percentages. Nonetheless, by day 90, animals receiving CS, CS+3p and CS+3p+TG02, accumulated less survival than the control PBS-vaccinated group. We also observed an enhancement of the tumor-growth in these groups, especially from day 40 and later (**Figure 2C**).

We analyzed the immune cell infiltrates in those tumors by flow cytometry. Tumors displayed heterogeneous levels of immune cells (CD45^+^), regardless their vaccination group (**Figure 2D**). Attending to the diverse immune subtypes, no major differences were found, excepting for an increment on T regulatory cells (Tregs) (CD4^+^ Foxp3^+^) and on the total percentage of CD4^+^ T cells in the group receiving the combined vaccine CS+3p+TG02. Although no differences among groups were observed in the percentage of immune cells expressing MHC-II, we also analyzed the expression levels of that marker. By comparing the median fluorescence intensity of MHC-II in the CD45^+^ MHC-II positive population, we discovered that group CS+TG02+3p had statistically significant lower MHC-II expression compared to the other vaccination groups.

The analysis of the systemic immune response generated by those vaccines in mice-bearing tumors showed that specific anti-Krasm IgGs in the serum were only found 10 days after the 3^rd^ boost, mostly in the CS+3p+TG02 group (**Supplementary Figure S5B**), although their levels were not statistically significant compared with those obtained in the other groups. After the 2^nd^ boost or at sacrifice time (**Supplementary Figures S5A–C**, respectively), no increment in the level of anti-Krasm specific IgGs were detected. Moreover, no differences in the spleen immune T cell populations were detected after incubation with antigens for 48 hours (**Supplementary Figure S5D**).

### 3.5 *In vitro* effect of CS NCs on pancreatic tumoral cells and innate immune cells

Our CS-Krasm vaccines containing IMQ as adjuvant, not only failed to prevent or reduce tumor growth *in vivo*, but they seemed to promote it. This effect was associated with a detected increase in the percentage of tumor infiltrating Tregs in the mouse group vaccinated with CS+3p+TG02.

**Figure 3:**
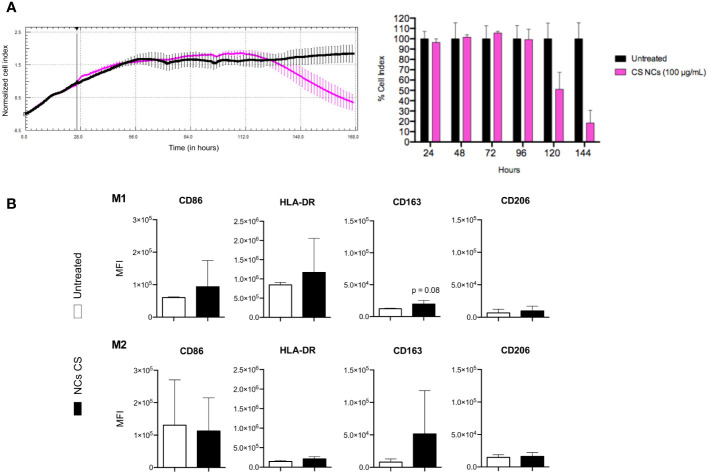
Effect of CS NCs in tumor cells in vitro. **(A)** Kinetics of the normalized cell index of ATQ303G in the presence of the CS NCs (left) and the cell index quantification (right). Untreated cells (black color) were used as control. **(B)** Modulation of marker expression on THP-1 cells differentiated to M1 and M2 after incubation with RMPI culture medium (white color) or CS NCs (black color) at 100 µg/mL for 24 h. The values are normalized to those obtained for the differentiated M1 and M2. For differentiation to M1, THP-1 cells were incubated for 48 h with 20 ng/mL IFN-γ + 5 ng/mL LPS + 10 ng/mL of GM-CSF, while for M2 they were incubated with 20 ng/mL IL-4 + 10 ng/mL M-CSF.

We attempted to decipher the mechanisms that could be acting to promote this *in vivo* tumor growth. We studied the effect of CS NCs on the mouse tumoral pancreatic ATQ303G cell line (K-Ras G12V and knock out for p53), using the xCELLigence system. We found an increase in the Cell Index during 50-72 hours of incubation, in both untreated and incubated with CS NCs cells, being maintained for approximately another 24 hours (**Figure 3A**). After 5 days, tumoral cells treated with CS NCs had less viability than untreated cells, pointing towards a cytotoxic effect of the CS NCs on the tumoral cells.

Then, we decided to study the potential involvement of the innate cell population. Tumor immune microenvironment is known to be extremely important in a tumor’s fate. Diverse signals and materials can modulate, for instance, the macrophage phenotype, polarizing them into M1 (pro-inflammatory - anti-tumoral phenotype) or M2 type (anti-inflammatory - repair - pro-tumoral phenotype).

We studied the effect of CS NCs on previously differentiated and polarized M1 or M2 macrophages, analyzing the expression of some markers mainly associated to M1 (Major histocompatibility complex), or M2 profiles [CD163 is a scavenger receptor used as a typical marker for M2 macrophages (19)]. We observed a tendency of a higher expression of the CD163 marker in both types of polarized macrophages (**Figure 3B**). No prominent differences in other markers were found.

We asked then whether CS NCs could be inducing mouse innate trained immunity, but in this case by modulating tolerance (favoring therefore tumor growth). We performed an *in vitro* assay using peritoneal macrophages collected from C57BL/6 mice. We incubated the macrophages overnight with CS nanocapsules, and after 7 days, the cells were exposed to LPS to assess their pro-inflammatory response through TNF-α quantitation (**Supplementary Figure S6A**). Macrophages previously incubated with CS NCs produced slightly lower levels of TNF-α (363.23 pg/mL mean) than naïve macrophages (434.24 pg/mL mean), but differences were not statistically significant (**Supplementary Figure S6B**). We verified that all samples shared similar levels of cell viability at the final time-point (**Supplementary Figure S6C**). Thus, the hypothesis that CS nanocapsules could induce tolerance through innate trained immunity was not confirmed by the *in vitro* approach.

The pro-tumoral effect induced by the CS NCs nanovaccines seemed to appear mostly *in vivo*, either used before and after tumor induction. No clear evidence was found in this study about the mechanisms underlying this effect, except that CS NCs are promoting an immunosuppressive response, including an augment of T regulatory cells within the tumor environment in the therapeutic vaccination with CS+TG02+3p or that CS-NCs are inducing a slight promotion of the macrophage polarization towards M2 phenotype.

### 3.6 Pro-tumorigenic effect of prophylactic inulin/polyarginine-Krasm vaccine

To assess whether the observed effects were due to the polymeric composition of the CS-NCs, we replaced the polymeric composition of the nanocapsules by designing another nanovaccine based on inulin and polyarginine shell (INU/pArg NCs). Both polymers have been proved to be biocompatible, safe and with adjuvant properties (20, 21). The complete INU/pArg nanovaccine also carried IMQ adjuvant in the lipidic core and antigens (our Krasm peptides or the TG02 peptides) adsorbed on the surface (**Supplementary Figure S7A**).

The INU/pArg NCs were characterised (**Supplementary Table S5**), but they proved to be less cytocompatible and hemocompatible than CS NCs (**Supplementary Figures S7B–D**). However, they demonstrated some immunogenic properties, based on their induction of ROS and secretion of cytokines (mainly INF-γ) (10).

Then, we tested the prophylactic effect of the INU/pArg-Krasm vaccines. Mice received three doses of either PBS, INU/pArg alone, INU/pArg+3p, INU/pArg+TG02 or INU/pArg+3p+TG02. After eight days, tumors were induced following the same scheme depicted in **Supplementary Figure S3A**. In this case, mice vaccinated with the INU/pArg nanocapsules (without peptides) displayed the lowest survival rates, with all individuals deceased by day 66 (**Supplementary Figure S6E**). Again, all the remaining vaccinated groups had less survival percentage than the control group (receiving PBS) by the end of the experiment.

These results indicate a detrimental effect of IMQ adjuvant in PDAC cancer vaccines. The pro-tumoral effect must be induced systemically, probably involving the immune response, as the *in vitro* incubation of the tumor cell line with INU/pArg NCs displayed a clear anti-tumoral effect (**Supplementary Figure S6F**).

## 4 Discussion

PDAC is a highly aggressive and lethal cancer with poor prognosis, with a growing incidence in many countries. The poor outcome is due to several factors like a delay in diagnosis (impending surgery), but also to some special PDAC characteristics including low vascularization and dense stroma and immune-suppressor microenvironment. These altogether lead to multidrug resistance capacity. There is a clear urgency for the development of new and effective therapies, as it is estimated that pancreatic cancer will double its associated mortality in the next 40 years (22).

In this work, we designed a new cancer vaccine that targets the K-Ras protein mutated at the G12V position, composed by CS nanocapsules with IMQ as adjuvant. The underlying rationale was that CS nanoparticles had previously showed good results as vaccines in several anti-tumoral therapies, especially as drug-delivery systems (23–25). Moreover, IMQ is widely used as an immunostimulant in skin cancer treatments.

We first assessed the effect of our vaccines in healthy mice. Mutations in the 12 codon of K-Ras have been described to induce specific CD8^+^ and CD4^+^ T-cell immune responses (26, 27). We designed three peptides of K-Ras containing G12V mutation to be presented in either MHC class I or class II molecules. We tested two peptide combinations: CS+2p, containing peptides p1 and p2, that should be presented *via* class II MHC, and CS+3p, containing three peptides (p1, p2 and p3) designed to be presented by both MHC class I and II. Both vaccines incremented the percentage of effector CD4^+^ and CD8^+^ T lymphocytes cells, losing the expression of the CD62L marker. Importantly, the vaccines also induced specific antibodies targeting the K-Ras G12V mutation. In both cases, better results were achieved with the combination of the three peptides (CS+3p group).

Furthermore, splenocytes of mice vaccinated with CS+3p secreted higher levels of some Th1 pro-inflammatory cytokines such as IFN-γ, GM-CSF, IL-2 or TNF-α, following antigen stimulation. However, we also reported the secretion of some immunomodulatory Th2 cytokines, including IL-4 or IL-10, and IL-6.

After the promising *in vitro* results, especially with the CS+3p combination, we assessed the tumor efficacy *in vivo*. We first tested its potential as a prophylactic vaccine. Currently, in the oncology field, there are only two preventive vaccines authorized for clinical use: one against papillomavirus-associated cervical cancer (28) and another against hepatitis B virus-associated hepatocellular carcinoma (29).

At short term, our preventive vaccination with the CS+3p prototype was successful, achieving a good survival rate (>60%) up to 45 days after tumor induction. However, when the study was prolonged beyond this time, there was an increase in mortality of all groups immunized with CS NCs. In these mice, we did not find K-Ras peptide-specific IgG antibodies in the blood, probably because their disappearance with time. We could not find neither any differences in the immune splenocyte populations after antigen stimulation *in vitro*.

No therapeutic efficacy associated with vaccination was observed neither when the tumors pre-existed at the beginning of the immunization regimen. Again, all groups vaccinated with CS NCs showed less survival rates than the PBS control group, having the CS+3p+TG02 combination the worst long-term outcome. It is worthy to indicate that we found an increase on the percentage of tumor-infiltrating Tregs in this group of mice receiving CS+3p+TG02, although no relevant differences in the immune populations were observed in the spleen.

Despite our encouraging first results, our vaccines seemed to induce a pro-tumoral effect in PDAC tumor-bearing mice, especially in the long-term, similarly to what it has been described for another K-Ras vaccine that favored tumor growth in skin cancer (in this case with a G12R mutation) (30). In this work, tumors grew faster and became much larger in the vaccinated mouse group. The authors could not clarify the mechanisms underlying this proliferation, but they proposed that an insufficient immune response could be stimulating the tumor growth, rather than inhibiting it (31, 32).

In our case, the increase of Tregs infiltrating the tumors of CS+3p+TG02-vaccinated mice could cause an immunosuppressive and tolerogenic effect helping tumor progression. The increase of Tregs after immunization with peptide vaccines together with cytokines (GM-CSF or IL-2), was also described in patients with different types of cancer carrying a mutated Ras (33). It is described that Tregs play a significant role in self-tolerance as well as in so-called immunosurveillance (34, 35). In addition, they are a fundamental part of the immunosuppressive microenvironment which is a distinctive trait of many cancers including PDAC (36). Moreover, this agrees with the observed high IL-10 production, the main cytokine produced by Tregs.

The second significant finding was the decrease of MHC-II expression, but not a decrease in the number of MHC-II-positive cells. An important mechanism of tumor scape is to impede maturation of dendritic cells (DCs), being immature DCs characterized by a lower expression of MHC class II molecule (37). Our observations suggest that vaccination with CS+3p+TG02 could be favoring the accumulation of immature DCs in the tumor environment, which might be contributing to a tolerogenic immune response. However, further analysis should be performed to characterise the antigen-presenting cells within the tumor and their functions.

Chitosan nanoparticles have been previously proven by our group and others to be good candidates in anti-pathogen vaccine designs (10, 38) or as a drug delivery system in cancer therapy (reviewed in (39)). However, their direct effects on tumor cells have been controversial, with several works reporting either anti (25, 40, 41) or pro-tumorigenic properties (42). Some of these pro-tumoral effects could be through their capacity to activate complement, promoting tumorigenesis and cancer progression (43). Despite the variability found by the cell differentiation model itself, it could be indicated that CS NCs favour an anti-inflammatory environment. Various studies corroborate our results, showing how CS in different forms is capable of inducing a polarization of macrophages towards an anti-inflammatory M2 phenotype (44, 45, 46).

In our hands, the *in vitro* incubation of tumoral cells with the CS NCs or their individual components did not induce significant tumoral growth at long-term. Although a certain increase in tumor growth and viability was observed within the first 48 hours, it is unlikely to affect the *in vivo* assay, since the nanovaccine was injected at the opposite flank of the tumor. Furthermore, in both *in vivo* experiments (prophylactic or therapeutic), we observed differences depending on the type of peptides (+3p, +TG02 or +3p+TG02) included on the CS NCs, pointing towards the specific action of the immune response. As far as we know, this is the first time that CS NCs have been tested in a pancreatic cancer vaccine.

An important factor to reckon in this prototype is the use of mutated self-antigens, instead of foreign antigens from infectious pathogens (10). This, altogether with a small peptide size and an insufficient immunostimulation, could deviate the immune response towards a tolerogenic state, as proposed by Siegel et al. (30). We also do not know whether the length of the peptides (9-15 mers) and their quantity, could affect antigen presentation (intracellular loading or direct binding) and how that affects to the subsequent immune activation.

We have previously described that CS NCs loaded with IMQ, but not empty CS NCs, induce the production of IL-10, IL-6 and TNF-α in mouse peritoneal macrophages in a dose dependent manner (9). These three cytokines were also found in the supernatant of splenocytes from those mice vaccinated with the CS+3p combination, along with other cytokines. It has been described that under specific circumstances, IL-6 and IL-10 could favor cancer development (47). We wondered then whether the pro-tumorigenic effect might be shaped by IMQ.

When we replaced the polymeric CS shell of the nanovaccine for INU/pArg, maintaining IMQ inside the core, immunization with our preventive vaccines also enhanced the subcutaneous tumor growth. These results support the hypothesis that the pro-tumoral effect might be influenced by IMQ. However, IMQ has been described to induce anti-tumoral effects when directly used (48–50) or loaded inside some nanocapsules (51), and it shows good immunogenic properties as vaccine adjuvant (11, 12). Nevertheless, it has also been observed that it could produce the opposite effect by favouring the production of IL-10 and Treg cell stimulation (13). This, altogether with our results, exposes a warning for the use of IMQ for cancer therapies, although further studies are needed to understand completely its mechanism of action. It is also important to perform experiments at short and long term (>60 days), because, as shown in this work, an anti-tumoral effect induced by a vaccine at short time, can be modified to generate negative effects months later.

Overall, the presence of Tregs infiltrating the tumor, the reduction of MHC-II expression, as well as the slight polarization of the macrophages *in vitro* towards an anti-inflammatory phenotype, support that our vaccines may exert a tolerogenic effect. However, Tregs were only found in one of the CS NCs-vaccinated group.

In the immunogenicity study using healthy mice, our vaccines seemed to induce a good immune response in both, lymphocyte cell activation and generation of specific antibodies recognizing mutated K-Ras, shortly after immunization. Thus, immunization with our vaccines did not directly induce T cell tolerance, but it may lead to a long-term insufficient response that promotes tumor growth. Because of that, we propose that the vaccine could be producing a dual effect, very much affected by the tumor environment. This idea is supported by the facts that in tumor-bearing mice vaccinated with CS+3p, less specific antibodies, decreased MHC-II expression and more Tregs infiltrating the tumor were found.

The mechanisms underlying the immunosuppression induced by our NCs in the pancreatic tumor remain to be solved. We propose that the effect might be driven by a combination of factors, being IMQ an important participant.

Our results open the way to further explore many factors that could contribute to this pro-tumorogenic results. We mainly focused on the lymphocyte response (activation, surface markers and specific antibody production), but there are other cell populations with the ability to induce tolerance *in vivo* (e.g., immature dendritic cells, myeloid derived suppressor cells) that remain to be characterised in further detail. If other concentrations of antigen or adjuvants will affect the outcome, also remain as appealing and unsolved questions. New prototypes combining CS or INU/pArg nanocapsules with different adjuvants (such as Poly I:C, MPLA, AS04, CpG oligonucleotides or GM-CSF) could be tested to clarify the adjuvant contribution to the tolerogenic situation. The effect of our prototypes in other types of cancers carrying G12V KRAS mutations also remains to be tested, maybe leading to a better prognosis in less immunosuppressive environments. This could help to understand why PDAC is so special regarding its absence of response to many different drugs or to the monoclonal antibodies against the immune check point inhibitors. Moreover, the ability of CS NCs to polarize macrophages into M2 phenotype must be an issue of concern in the design of future anti-tumoral vaccines.

## 5 Conclusions

Our prophylactic vaccine against pancreatic cancer (based on CS NCs, IMQ and containing specific mutated K-Ras peptides at G12V position), showed certain efficacy at short time. However, at long-term it showed detrimental pro-tumorigenic effects, used either as a preventive or therapeutic treatment. Another anti-K-Ras vaccine, TG02, using GM-CSF as adjuvant, showed the best survival data in both cases. We observed that our NCs exerted a tolerogenic effect by inducing a slight re-polarization of pro-inflammatory macrophages M1 towards an anti-inflammatory M2 phenotype and an increment on the tumor infiltrating Tregs in some mice. Tumor growth also increased after vaccination with similar NCs based on INU/pArg, which included IMQ as adjuvant too.

Although we could not completely decipher yet the mechanisms that promoted the tumor growth, the observations call for caution in the use of IMQ and CS or INU/pArg nanocapsules in PDAC cancer therapy.

## Data availability statement

The raw data supporting the conclusions of this article will be made available by the authors, without undue reservation.

## Ethics statement

The studies involving human participants were reviewed and approved by CEEA Xunta de Galicia, code 2018/269. The patients/participants provided their written informed consent to participate in this study. The animal study was reviewed and approved by CEEA Xunta de Galicia, code ES360570215601/18/INV MED 02/CANC.[3]/C. Experiment done at i3S was approved by the institutional board responsible for animal welfare (ORBEA) and authorization was issued by the competent Portuguese authority (DGAV), code 014036/2019-07-24.

## Author contributions

AM-P, LD-G and AC performed the experiments and analyzed the data. MV and AC provided counselling. RS-V and AG-F conceived the study and contributed with funding acquisition. AM-P wrote the paper with input from all other authors. All the authors have read and approved the manuscript.

## Supplementary material

Supplementary Figure 1 Physicochemical and biological characterization of CS-nanoparticles. **(A)** Polymeric nanovaccine prototype against the mutated protein K-Ras G12V. **(B)** Cellular apoptosis assay after 24 hours of incubation with CS NCs at 50 and 100 µg/mL in Jurkat and hPMBCs. One-way ANOVA and Dunnett’s multiple comparison tests were used for statistical analysis. **(C)** Percentage of hemolysis induced by CS NCs in mouse and human blood. The NCs were tested at different concentrations (25, 50 and 100 µg/mL). PBS and 1% Triton X-100 were used as negative and positive control, respectively. **(D)** Determination of Prothrombin time (PT), Activated partial thromboplastin time (APTT) and Thrombin time (TT) after incubation of plasma with CS NCs at three different concentrations (25, 50 and 100 µg/mL). Mann-Whitney test was used for statistical analysis comparing to PBS negative control. *p < 0.05.

Supplementary Figure 2 Lymphocyte biomarkers in splenocytes from vaccinated mice, left unstimulated or after stimulation with individual Krasm peptides. Vaccination regimen is depicted in **Figure 1A**. Ten days after 3^rd^ immunization spleens were collected and cultured. Splenocytes were left unstimulated or incubated with the individual Krasm peptides (peptide 1, peptide 2 or peptide 3) for 72 hours. Then, lymphocyte biomarkers were evaluated by flow cytometry in CD4^+^ T **(A)** and CD8^+^ T cell **(B)** populations. The Kruskal–Wallis test and Dunn’s multiple comparison test were used for statistical analysis. *p < 0.05.

Supplementary Figure 3 Effect of CS-Krasm used as preventive-vaccination in tumor-bearing mice. **(A)** Experimental protocol is depicted. C57BL/6 mice 8-weeks old were vaccinated three times at 21-days intervals (n=5 mice per group). Eight days after last boost, mice were subcutaneously injected ATQ303G pancreatic cells in the left flank. Whenever mice reached their ethical time-point, mice were sacrificed, with blood and spleens collected. **(B)** Mice percentage of survival from day 0 (tumor injection) to day 110. **(C)** The levels of specific IgGs in serum that recognize a mixture of Krasm peptides (peptide 1, peptide 2 and peptide 3) at their readout time-point were determined by ELISA immunoassay. **(D)** Splenocytes were stimulated for 48 hours. CS+3p group was incubated with the 3-peptide-mixture; TG02 and CS+TG02 groups were incubated with the TG02-peptide-mixture; and the remaining groups with the combination 3-peptide and TG02-peptide-mixtures. After that, lymphocyte biomarkers were evaluated by flow cytometry.

Supplementary Figure 4 Tumor volumes of vaccinated mice. C57BL/6 mice 8-weeks old were subcutaneously injected with ATQ303G pancreatic cells in the left flank. Changes in tumor volumes for each vaccination group are displayed throughout the duration of the experiment. When tumors reached a volume of 100-150 mm^3^, mice were vaccinated three times at 21-days intervals (n=10 per group). Whenever mice reached their ethical time-point, mice were sacrificed.

Supplementary Figure 5 Characterization of the immune systemic response generated by CS-Krasm therapeutic-vaccination in tumor-bearing mice. Experimental protocol is depicted in The levels of specific IgGs in serum that recognize a mixture of Krasm peptides (peptide 1, peptide 2 and peptide 3) were determined by ELISA immunoassay from serum collected ten days after 2^nd^ **(A)** and 3^rd^ **(B)** immunizations or at the readout **(C)** time-point. **(D)** Splenocytes were stimulated for 48 hours. CS+3p group was incubated with the 3-peptide-mixture; TG02 group was incubated with the TG02-peptide-mixture; and the remaining groups with both, 3-peptides and TG02-peptide-mixtures. After that, lymphocyte biomarkers were evaluated by flow cytometry.

Supplementary Figure 6 Trained-immunity effect of CS NCs in vitro. **(A)** Experimental protocol is depicted. Briefly, peritoneal macrophages from mice were incubated overnight with CS nanoparticles (day 0), washed and rest for 6 days. At day 7, LPS was added during 3 hours, cells were washed and supernatants were collected after overnight resting. **(B)** TNF-α levels at day 8 measured using ELISA assay. **(C)** Percentages of macrophage viability at day 8 measured using the MTS assay, which was performed as described in (10). Percentages are normalized to untreated control.

Supplementary Figure 7 Physicochemical and biological characterization of INU/pArg-nanoparticles and its effect as a preventive vaccination in tumor-bearing mice. **(A)** Polymeric INU/pArg nanovaccine prototype against the mutated protein K-Ras G12V. **(B)** Cellular apoptosis assay after 24 hours of incubation with INU/pArg NCs at 50 and 100 µg/mL in Jurkat and hPMBCs. One-way ANOVA and Dunnett’s multiple comparison tests were used for statistical analysis. *p < 0.05 compared to untreated cells. **(C)** Percentage of hemolysis induced by INU/pArg NCs in mouse and human blood. The NCs were tested at different concentrations (25, 50 and 100 µg/mL). PBS and 1% Triton X-100 were used as negative and positive control, respectively. **(D)** Determination of Prothrombin time (PT), Activated partial thromboplastin time (APTT) and Thrombin time (TT) after incubation of plasma with INU/pArg NCs at three different concentrations (25, 50 and 100 µg/mL). Mann-Whitney test was used for statistical analysis comparing to PBS negative control. *p < 0.05. **(E)** 8-weeks old C57BL/6 mice were vaccinated three times at 21-days intervals (n=5 mice per group), and eight days after the last boost they were subcutaneously injected ATQ303G pancreatic cells in the left flank. The graph depicts the percentage of survival from day 0 (tumor injection) to day 110.

Supplementary Table 1 Selected peptides using IEDB MHC binding prediction tool.

Supplementary Table 2 Summary of the times and volumes used for each reagent for the different coagulation tests.

Supplementary Table 3 List of antibodies used in flow cytometry - macrophage polarization.

Supplementary Table 4 List of antibodies and reagents used in flow cytometry – splenocytes and tumor analysis.

Supplementary Table 5 Physico-chemical characterization of nanocapsules of INU/pArg in the absence and presence of mutated antigenic peptides.
