# Supplementary material for: Immunization with nanovaccines containing mutated K-Ras peptides and imiquimod aggravates heterotopic pancreatic cancer induced in mice
Source: Front Immunol. 2023 Apr 12;14:1153724. doi: 10.3389/fimmu.2023.1153724 (PMC10130386; doi:10.3389/fimmu.2023.1153724)
Supplement: Supplementary Figure 1 — Physicochemical and biological characterization of CS-nanoparticles. (A) Polymeric nanovaccine prototype against the mutated protein K-Ras G12V. (B) Cellular apoptosis assay after 24 hours of incubation with CS NCs at 50 and 100 µg/mL in Jurkat and hPMBCs. One-way ANOVA and Dunnett’s multiple comparison tests were used for statistical analysis. (C) Percentage of hemolysis induced by CS NCs in mouse and human blood. The NCs were tested at different concentrations (25, 50 and 100 µg/mL). PBS and 1% Triton X-100 were used as negative and positive control, respectively. (D) Determination of Prothrombin time (PT), Activated partial thromboplastin time (APTT) and Thrombin time (TT) after incubation of plasma with CS NCs at three different concentrations (25, 50 and 100 µg/mL). Mann-Whitney test was used for statistical analysis comparing to PBS negative control. *p < 0.05. [file DataSheet_1.zip › Table S2.pdf]

Table S2. Summary of the times and volumes used for each reagent for the different coagulation tests.

	Equipment Parameters		Volumes		Normal coagulation time
	Maximum time	*Incubation time*	*Plasma and other reagents*	*Coagulation inducing reagents*	
PT	120 sec	180 sec	50 µL plasma + 50 µL de PTT-A	50 µL $CaCl_2$	≤ 34,1 sec
APTT	60 sec	60 sec	100 µL plasma	100 µL Trombin	≤ 21 sec
TT	60 sec	120 sec	100 µL plasma	100 µL Neoplastin	≤ 13,4 sec

APTT: activated partial thromboplastin time; $CaCl_2$: calcium chloride; PT: prothrombin time; PTT-A: activated partial thromboplastin time reagent; sec: seconds; TT: thrombin time.
