# Supplementary material for: Immunization with nanovaccines containing mutated K-Ras peptides and imiquimod aggravates heterotopic pancreatic cancer induced in mice
Source: Front Immunol. 2023 Apr 12;14:1153724. doi: 10.3389/fimmu.2023.1153724 (PMC10130386; doi:10.3389/fimmu.2023.1153724)
Supplement: Supplementary Figure 1 — Physicochemical and biological characterization of CS-nanoparticles. (A) Polymeric nanovaccine prototype against the mutated protein K-Ras G12V. (B) Cellular apoptosis assay after 24 hours of incubation with CS NCs at 50 and 100 µg/mL in Jurkat and hPMBCs. One-way ANOVA and Dunnett’s multiple comparison tests were used for statistical analysis. (C) Percentage of hemolysis induced by CS NCs in mouse and human blood. The NCs were tested at different concentrations (25, 50 and 100 µg/mL). PBS and 1% Triton X-100 were used as negative and positive control, respectively. (D) Determination of Prothrombin time (PT), Activated partial thromboplastin time (APTT) and Thrombin time (TT) after incubation of plasma with CS NCs at three different concentrations (25, 50 and 100 µg/mL). Mann-Whitney test was used for statistical analysis comparing to PBS negative control. *p < 0.05. [file DataSheet_1.zip › Table S3.pdf]

Table S3. List of antibodies used in flow cytometry - macrophage polarization

Antibody	Reference
CD206 PE-Cy7	Immunostep SL, 1399990019
CD163 BV421	BD, 562643
HLA-DR APC-Vio770	Miltenyi Biotec, 130-111-792
CD40 APC	Immunostep SL, 40A-100T
CD86 BB515	BD, 564544
CD14 PerCP	Immunostep SL, 14PP-100T
