# Supplementary material for: Immunization with nanovaccines containing mutated K-Ras peptides and imiquimod aggravates heterotopic pancreatic cancer induced in mice
Source: Front Immunol. 2023 Apr 12;14:1153724. doi: 10.3389/fimmu.2023.1153724 (PMC10130386; doi:10.3389/fimmu.2023.1153724)
Supplement: Supplementary Figure 1 — Physicochemical and biological characterization of CS-nanoparticles. (A) Polymeric nanovaccine prototype against the mutated protein K-Ras G12V. (B) Cellular apoptosis assay after 24 hours of incubation with CS NCs at 50 and 100 µg/mL in Jurkat and hPMBCs. One-way ANOVA and Dunnett’s multiple comparison tests were used for statistical analysis. (C) Percentage of hemolysis induced by CS NCs in mouse and human blood. The NCs were tested at different concentrations (25, 50 and 100 µg/mL). PBS and 1% Triton X-100 were used as negative and positive control, respectively. (D) Determination of Prothrombin time (PT), Activated partial thromboplastin time (APTT) and Thrombin time (TT) after incubation of plasma with CS NCs at three different concentrations (25, 50 and 100 µg/mL). Mann-Whitney test was used for statistical analysis comparing to PBS negative control. *p < 0.05. [file DataSheet_1.zip › Table S4.pdf]

Table S4. List of antibodies and reagents used in flow cytometry – splenocytes and tumor analysis

**Antibody**	**Reference**
CD3 PerCp5.5.	BD, 560527
CD4 APC H7	BD, 560181
CD8 PE-Cy7	BD, 552877
CD25 APC	BD, 558643
CD44 PE	Miltenyi 130-102-606
CD62L BV421	BD, 562910
Foxp3 Alexa Fluor 488	BD, 560403
IFN-γ BV650	BD, 563854
IL4 BV605	BD, 564007
CD45 VioBlue	Miltenyi, 130-110-664
MHC-II AF647	BD, 562367
NK1.1 PE	Miltenyi, 130-120-497
Viability Stain 510	BD, 564406
