# Supplementary material for: Immunization with nanovaccines containing mutated K-Ras peptides and imiquimod aggravates heterotopic pancreatic cancer induced in mice
Source: Front Immunol. 2023 Apr 12;14:1153724. doi: 10.3389/fimmu.2023.1153724 (PMC10130386; doi:10.3389/fimmu.2023.1153724)
Supplement: Supplementary Figure 1 — Physicochemical and biological characterization of CS-nanoparticles. (A) Polymeric nanovaccine prototype against the mutated protein K-Ras G12V. (B) Cellular apoptosis assay after 24 hours of incubation with CS NCs at 50 and 100 µg/mL in Jurkat and hPMBCs. One-way ANOVA and Dunnett’s multiple comparison tests were used for statistical analysis. (C) Percentage of hemolysis induced by CS NCs in mouse and human blood. The NCs were tested at different concentrations (25, 50 and 100 µg/mL). PBS and 1% Triton X-100 were used as negative and positive control, respectively. (D) Determination of Prothrombin time (PT), Activated partial thromboplastin time (APTT) and Thrombin time (TT) after incubation of plasma with CS NCs at three different concentrations (25, 50 and 100 µg/mL). Mann-Whitney test was used for statistical analysis comparing to PBS negative control. *p < 0.05. [file DataSheet_1.zip › Table S5.pdf]

Table S5. Physical-chemical characterization of nanocapsules of INU/pArg in the absence and presence of mutated antigenic peptides.

NCs	Size (nm)	ζ (mV)
INU/pArg	132.7 ± 3.5	(+) 21.9 ± 4.6
INU/pArg +3p	123.8 ± 0.4	(+) 14.6 ± 0.9
INU/pArg +TG02	129.6 ± 3.5	(+) 16.6 ± 0.4

INU/pArg: inulin/polyarginine; NCs: nanocapsules; TG02: Targovax vaccine; ζ: zeta potential; 3p: combination of the three mutated peptides K-Ras G12V
